# Chemokine Receptor-4 Targeted PET/CT Imaging with ^68^Ga-Pentixafor in Head and Neck Cancer—A Comparison with ^18^F-FDG and CXCR4 Immunohistochemistry

**DOI:** 10.3390/diagnostics14131375

**Published:** 2024-06-28

**Authors:** Bawinile Hadebe, Lerwine Harry, Lerato Gabela, Siphelele Masikane, Maryam Patel, Sizwe Zwane, Venesen Pillay, Presha Bipath, Nonhlanhla Cebekhulu, Nozipho Nyakale, Prathima Ramdass, Mpumelelo Msimang, Colleen Aldous, Mike Sathekge, Mariza Vorster

**Affiliations:** 1 Department of Nuclear Medicine, College of Health Sciences, University of KwaZulu Natal, Private Bag X54001, Durban 4001, South Africa; 2 Inkosi Albert Luthuli Central Hospital, Durban 4001, South Africa; 3 Department of Radiation Oncology, College of Health Sciences, University of KwaZulu Natal, Private Bag X03, Durban 4001, South Africa; 4 Department of Nuclear Medicine, Sefako Makgatho Health Science University, Pretoria 0208, South Africa; 5 Department of Nuclear Medicine, Jawaharlal Nehru Hospital, Rose Belle 51829, Mauritius; 6 Department of Anatomical Pathology, National Health Laboratory Service, Durban 4000, South Africa; 7 Department of Genetics, College of Health Sciences, University of KwaZulu Natal, Durban 4001, South Africa; 8 Department of Nuclear Medicine, Faculty of Health Sciences, University of Pretoria, Pretoria 0002, South Africa

**Keywords:** CXCR4, Pentixafor, PET, HIV, head and neck carcinoma

## Abstract

Background: Head and neck squamous cell carcinoma (HNSCC) is common, and its incidence is increasing, particularly in HIV-infected individuals who present with more aggressive disease. Despite aggressive treatment, the prognosis remains poor because of resistance to chemoradiation therapy. So far, studies report very low [^68^Ga]Ga-Pentixafor avidity in HNSCC. This study investigated the diagnostic performance of CXCR4-directed imaging of carcinoma of the oral cavity, oropharynx, and nasopharynx with positron emission tomography/computed tomography (PET/CT) using the radiolabelled chemokine ligand [^68^Ga]Ga-Pentixafor and explored its ability to quantify CXCR4 expression in vivo. Materials and Methods: In this prospective cross-sectional study, twenty-three (23) patients aged 52.9 ± 10.4 (19.6), 17 males and 6 females with primarily diagnosed (*n* = 17) or pre-treated (*n* = 6) SCC of the oral cavity (OCSCC, *n* = 11), oropharynx (OPSCC, *n* = 9), nasopharynx (NPSCC, *n* = 2) and unknown primary (*n* = 1) underwent imaging with [^68^Ga]Ga-Pentixafor-PET/CT. In 16/23 patients 2-[18F]fluoro-2-deoxy-D-glucose ([^18^F]F-FDG) served as a standard reference. All lesions were visually rated using a 5-point Likert scale. For both tracers, maximum standardized uptake values (SUVmax) and the total lesion uptake (TLU) were recorded and compared using the Wilcox-signed rank test. In addition, the tumor-to-background ratios were derived using the liver (TLR), spleen (TSR), and posterior cervical muscles (TMR) as background. The relationships between the SUVs of the two tracers were assessed using the Spearman correlation. CXCR4 immunohistochemistry (IHC) staining was correlated with ^68^Ga-Pentixafor-PET/CT in 21/23 patients. Results: Ninety-one percent (21/23) of tumors were visually detected on [^68^Ga]Ga-Pentixafor; however, [^68^Ga]Ga-Pentixafor was less intense compared with [^18^F]F-FDG-PET. Quantitative analysis showed higher [^18^F]F-FDG SUVmax in comparison with [^68^Ga]Ga-Pentixafor (16 ± 6.7 vs. 5.8 ± 2.6 g/mL, *p =* 0.011) and SUVmean (9.3 ± 4.1 vs. 3± 1.6 g/mL, *p <* 0.001) and TBR 4.9 ± 2.3 vs. 2.36 ± 1.4 *p =* 0.014. Nasopharyngeal cancer demonstrated more intense tracer accumulation than oropharyngeal and oral cavity malignancies. CXCR4 IHC staining was positive in 15/21 patients, and there was a statistically significant correlation between IHC staining and [^68^Ga]Ga-Pentixafor SUVmean r = 0.5 *p* = 0.027, and performance status r = 0.83 *p* = 0.0104. Conclusions: In conclusion, although [^68^Ga]Ga-Pentixafor cannot replace [^18^F]F-FDG as a diagnostic tool because of its lower avidity, the correlation between CXCR4 targeted ^68^Ga-Pentixafor PET imaging and CXCR4 IHC staining indicates the potential of ^68^Ga-Pentixafor as an effective tool for selecting patients who may benefit from therapies targeting CXCR4. In addition, [^68^Ga]Ga-Pentixafor has no physiological brown fat uptake, which often obscures cervical lesions on [^18^F]F-FDG PET/CT imaging.

## 1. Introduction

Squamous cell carcinoma of the head and neck (HNSCC) is a serious health problem globally and is the sixth most common cancer worldwide, with 890,000 new cases and 450,000 deaths in 2022 [1]. Despite advances in treatment such as robotic surgery and new chemotherapeutic drugs, the morbidity and mortality associated with oral cavity and oropharyngeal cancer is still significant as most patients present late with locally advanced unresectable disease with a 5-year survival rate of 50% [2]. The incidence of HNSCC is rising, particularly in HIV-infected communities. Tumor recurrence is seen in 15–50% of patients with HNSCC, mainly due to resistance and insufficient radiation dose [3]. As a result, attempts have been made to understand tumor biology better, and there has been a rising interest in the development of less toxic, targeted therapies for this condition.

Chemokine receptor 4 (CXCR4) is overexpressed in the tumor microenvironment of more than 20 malignancies, including HNSCC [4]. Its expression is related to larger tumor size, angiogenesis, and the development of metastasis [5]. Its ligand CXCL12 is abundant in organs such as the liver, lymph nodes, and bone, and CXCR4 activation leads to the migration of cancer cells toward these organs, leading to metastasis in these areas [6]. The CXCR4−CXCL12 axis also provides a protective niche for tumor cells in the bone marrow, which protects cancer cells from chemotherapy [7]. Qiao et al. found significantly higher CXCR4 expression in high-grade tumors compared with low-grade tumors and significant suppression of apoptosis [8].

Several CXCR4 antagonists have been tested using HNSCC cell lines, and the results show that antagonism of CXCR4/CXCR7-CXCL12 downregulates the expression of the chemokines axis and, therefore, could be used to control and potentially even cure nasopharyngeal cancer [8]. CXCR4 antagonist, AMD3100, decreases cell migration and cell invasion of oral cancers and inhibits lymph node metastasis in these cells due to the reduced migration of tumor cells by the suppression of the SDF-1/CXCR4 gradient and inhibition of lymphangiogenesis [9]. In addition, Uchida et al. investigated another CXCR4 inhibitor, AMD07, and found that AMD07 significantly prevented the metastasis of oral cancer cells to the lungs of nude mice [10].

[^68^Ga]Ga-Pentixafor has been identified as a molecular probe for non-invasive measurement of CXCR4 expression in tumors, and the limited available literature so far has shown low-grade [^68^Ga]Ga-Pentixafor uptake in HNSCC [11,12,13].

In this context, [^68^Ga]Ga-Pentixafor could potentially upstage patients by identifying brain metastasis not seen on [^18^F]F-FDG and possibly enable better patient stratification and prognosis. Also, [^18^F]F-FDG is limited by the presence of brown fat uptake, which may obscure lesions, particularly in the neck. Thus, the aim of this pilot study was to explore the utility of [^68^Ga]Ga-Pentixafor in patients with cancer of the head and neck and to compare this novel tracer with [^18^F]F-FDG on PET/CT imaging as well as CXCR4 immunohistochemistry staining.

## 2. Materials and Methods

### 2.1. Study Population

**Figure 1:**
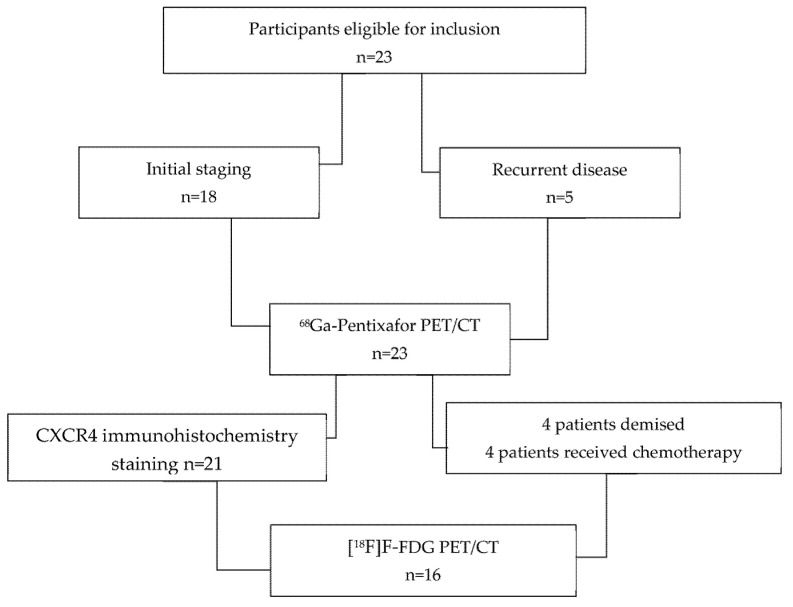
Flowchart displaying the composition of enrolled patients.

Twenty-three patients with histologically confirmed locally advanced carcinoma of the oral cavity, nasopharynx, and oropharynx were prospectively recruited into this study. Inclusion criteria were age ≥ 18 years, biopsy-proven head and neck carcinoma, and a signed consent form. Exclusion criteria included technically suboptimal scans and a synchronous malignancy. The patients were referred for PET/CT imaging as part of their work-up for initial staging prior to therapy (*n* = 18) or suspected recurrence (*n* = 5); all 23 patients underwent [^68^Ga]Ga-Pentixafor PET/CT. A total of 4 patients died before the [^18^F]F-FDG PET/CT appointment; 3 patients had chemotherapy in the interim, and therefore, their [^18^F]F-FDG PET/CT scans were not included in the analysis. The composition of enrolled patients is summarized in Figure 1. Informed consent was obtained from the patients for the scan and for accessing their hospital records. This study was approved by the Human Research Ethics Committee of the University of KwaZulu Natal (protocol reference number: BREC/00003636/2021). All procedures were performed in accordance with the ethical standards of the institutional research committee and in alignment with the 1964 Helsinki Declaration and its later amendments.

### 2.2. [^68^Ga]Ga-Pentixafor Synthesis

The synthesis of [^68^Ga]Ga-Pentixafor was performed in a semi-automated, GMP-compliant procedure using a IQS ^®^ module (ITG Isotope Technologies, Garching, Germany) equipped with a disposable single-use cassette kit (ABX, Germany). The eluate (^68^Ga^3+^ in 0.6 M HCl) of a ^68^Ge/^68^Ga-generator (ITM Medical Isotopes, Garching/Munich, Germany) was transferred to a cation exchange cartridge, eluted with 5 mls NaCl, added to a solution of 40 µg Pentixafor (PentixaPharm, Berlin, Germany) in HEPES-buffer and heated for 6 min at 105 °C. The product was immobilized on a SepPak C18 cartridge, washed with water, and eluted with ethanol/water 50/50. The eluate was passed through a sterile filter (0.22 µm) into a sterile vial and diluted with phosphate buffer solution to a total volume of 15 mL. Radiochemical purity was determined by thin-layer chromatography using 0.1 M ammonium acetate. Radiochemical yields of the prepared derivatives were found to be >95% for all the derivatives. The specific activity ranged from 30–65 GBq/mmol.

### 2.3. [^68^Ga]Ga-Pentixafor PET/CT Imaging Procedure

There was no specific patient preparation for the [^68^Ga]Ga-Pentixafor PET/CT scan. The injected activity of ^68^Ga-Pentixafor was (1.4–4 MBq/kg) and ranged between 78–210 MBq. We obtained whole body (vertex to mid-thigh) PET/CT images at 60 min post tracer injection. PET imaging was acquired in 3D mode at 7 min per bed position. We used CT data for attenuation correction and anatomic delineation of lesions. We performed image reconstruction using TruX + TOF (ultraHD-PET) (two iterations, 21 subsets) followed by post-reconstruction filtering with a Gaussian filter applied at 5.0 mm FWHM.

### 2.4. [^18^F]F-FDG PET/CT Imaging Procedure

Patient preparation for [^18^F]F-FDG PET/CT included a minimum of 4 h of fasting as per the published guidelines. Blood glucose before [^18^F]F-FDG injections was less than 7 mmol/L in all cases. The injected activity of [^18^F]F-FDG was between 2–4 MBq/kg. We imaged patients at 60 min post injection. All patients were imaged on a Biograph mCT PET/CT scanner equipped with a 64-slice CT (Siemens Medical Solutions, Lincolnshire, IL, USA). A vertex to mid-thigh CT scan was performed with parameters adjusted for patients’ weight (120 KeV, 40–150 mAs) with a section width of 5 mm and pitch of 0.8.

### 2.5. Image Analysis

Two experienced nuclear medicine physicians reviewed the [^68^Ga]Ga-Pentixafor PET/CT images. Reconstructed images were displayed as maximum intensity projection images, PET, CT, and fused PET/CT in the axial, coronal, and sagittal planes on a dedicated workstation equipped with syngo via software version 8.7 (Siemens Medical Solutions, Lincolnshire, IL, USA).

### 2.6. Qualitative Analysis

The 60-min whole body images were analyzed for bio-distribution and qualitative assessment of tumor uptake. We performed a qualitative assessment of the images and recorded our findings using a computer-generated 5-point Likert scale (similar to the Deauville criteria) with 1 = no uptake, 2 = uptake less or equal to normal blood pool (aorta), 3 = uptake more than blood pool similar but less than the liver, 4 = focal uptake moderately higher than the liver, and 5 = uptake markedly above liver activity [14].

### 2.7. Semi-Quantitative Analysis

For both tracers, images were analyzed for the presence of tracer accumulation in the primary tumor, nodal, and distant metastases. Nodal metastasis was differentiated from inflammation based on the pattern of uptake and CT characteristics.

Semi-quantitative analyses were performed using a volume of interest (VOI) placed over target lesions with an isocontour threshold of 40%. The maximum standardized uptake value (SUVmax), mean standardized uptake value (SUVmean), total lesion uptake (TLU), tumor-to-muscle ratio (TMR), as well as tumor-to-liver background ratio (TLR), and tumor-to-blood pool ratio (TBR) were recorded. TLU refers to the product of mean SUV and MTV, while MTV represents the size of tumor tissue that is actively taking up ^18^F-FDG. The semispinalis muscle was used for the calculation of the TMR, a 1.5 cm^3^ VOI placed over the liver was used for the TLR, and the thoracic aorta for the TBR.

### 2.8. Histopathological Analysis

CXCR4 immunohistochemistry was performed at the National Health Laboratory Service (NHLS) Laboratory at Inkosi Albert Luthuli Central Hospital. CXCR4 staining was categorised according to the intensity and percentage of stained cells. Overall staining intensity for CXCR4 was scored into four categories as follows: 0 (absence of staining), 1 (mild), 2 (moderate), 3 (strongly positive). The percentage of stained cells was also recorded.

Immunostaining of p16 was also performed as a surrogate marker for human papillomavirus (HPV). The proliferative index of the tumor (Ki67) staining was also performed on available tumor tissue, and the percentage staining was compared with [68Ga]Ga-Pentixafor PET/CT metrics on imaging and survival.

### 2.9. Statistical Analysis

The statistical data analysis was conducted using the R Statistical computing software of the R Core Team, 2020, version 3.6.3. The results were presented in the form of descriptive and inferential statistics. Where applicable, the descriptive statistics of numerical measurements were summarized as the minimum, maximum, quartiles, interquartile range, means, standard deviation, and coefficient of variation. Multidimensional numerical variables were presented as correlation plots, and the associations were assessed using correlation tests. Depending on the distribution of the numerical variables between two independent groups, mean or median differences were assessed using either a *t*-test or Wilcoxon, respectively. All the inferential statistical analysis tests were conducted at 5% levels of significance. Some other results were provided as descriptive statistics only, owing to our relatively small sample size.

## 3. Results

Demographic details:

Detailed patient characteristics (17 males and 6 females, mean age 52, standard deviation 10, range 35–73 years) are presented in Table 1. Sixteen patients were black (65%), 4 (17%) Indian, and 4 (17%) caucasian.

Clinical findings:

All patients had locally advanced stage III (*n* = 4), stage IVb (*n* = 12), and stage IVc (*n* = 4) disease. Histological grading showed 20 patients with moderately differentiated SCC, 1 patient with poorly differentiated SCC, 1 patient with undifferentiated SCC, 1 patient with mucoepidermoid carcinoma, and 1 patient with myoepithelial carcinoma. Three patients underwent [^68^Ga]Ga-Pentixafor PET/CT as part of restaging after radiotherapy (#6, 15) completed 8 and 6 months prior to imaging or chemoradiotherapy (#2) completed 3 months prior; and patient # 16 underwent [^68^Ga]Ga-Pentixafor PET for evaluation of suspected recurrence after chemotherapy performed 3 months prior. In all cases, no intervention (e.g., chemotherapy or surgery) was performed between the two scans.

A total of nine patients (36%) had SCC of the oropharynx, 11 patients (50%) had SCC of the oral cavity, and 2 patients (9%) had nasopharyngeal SCC, and in 1 (4,5%) patient, the primary was not detected. A total of 90 (90%) (19/23) patients had lymph cervical node metastasis, 2 patients had limited disease, and 2 of the patients had distant metastasis to the lungs and liver. Fourteen percent (4/23) of patients were HIV positive. Eleven (48%) of the study participants had died at the time of the analysis.

^68^Ga-Pentixafor-PET findings

A total of 21 of the 23 (91%) patients had visually detectable disease on the [^68^Ga]Ga-Pentixafor-PET. The primary lesions exhibited a median SUVmax of 5.01 ranging between 3.01–11.73, as shown in Table 2. and median TBR of 1.97 (Q1–Q3 1.58–2.75) and median TMR 3.92 (Q1–Q3 3.25–4.89). Interestingly, 5 of the 17 patients (19%) demonstrated visually more avid accumulation of ^68^Ga-Pentixafor in lymph nodes compared with the primary lesion (patients # 3,5,10,11,22) with SUVmax primary 5.01, 3.74, 5.61, and 8.41 vs. 4.61, 7.79, 5.71, and 7.98, respectively, on the lymph nodes.

### 3.1. Correlation of Pentixafor with Clinical Findings

Forty-five (45%) of the participants had low hemoglobin. There was a moderate negative correlation between [^68^Ga]Ga-Pentixafor total lesion uptake (TLU) and the hemoglobin (Hb), correlation efficient *r =* −0.5, *p* = 0.029.

Seventeen percent (17%) 4/23 of the patients were HIV positive; there was a higher accumulation of both [^68^Ga]Ga-Pentixafor and [^18^F]F-FDG in HIV-positive patients. [^68^Ga]Ga-Pentixafor total lesion uptake (TLU) was higher in HIV-infected HN cancer patients with a median TLU of 470 (303–473) in HIV-positive vs. 65.8 (43.2–108) in HIV-negative patients *p =* 0.001. Also, the [^68^Ga]Ga-Pentixafor TBR was higher at 3.20 ± 0.5 in HIV-positive patients compared with 2.43 ± 0.58 in the HIV-negative patients, *p =* 0.051. There was higher [^68^Ga]Ga-Pentixafor SUVmax in the HIV-positive patients SUVmax 7.40 ± 3.77 compared with 4.96 (3.48–8.26) in the HIV-negative patients *p =* 0.323, although this did not reach statistical significance. The [^18^F]F-FDG parametric measurements were also higher in the HIV-positive patients median SUVmax 19.5 (Q1–Q3) 17.4–20.6 compared with 13.0 (9.96–17.6) in HIV-negative patients, although this was not statistically significant *p* = 0.119.

### 3.2. Comparison of FDG and ^68^Ga-Pentixafor

**Figure 2:**
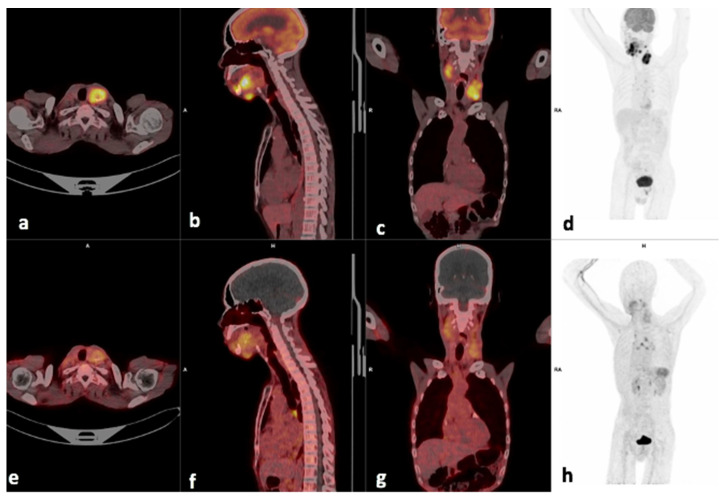
Example of a moderate CXCR4-positive, high FDG-positive patient. Display of [^18^F]F-FDG-PET/CT (**a**–**d**) and ^68^Ga]Pentixafor-PET (**e**–**h**) (interval between both scans, 14 days) in a 60-year-old male patient with oral cavity SCC (patient #6). [^68^Ga]Pentixafor-PET demonstrates less intense tracer accumulation at the site of primary SUVmax 4.84 (**b**) compared with 18.36 on [^18^F]F-FDG (**f**), the left cervical node metastasis also showed more intense uptake on [^18^F]F-FDG (**c**), SUVmax 14.41 versus 5.29 on [^68^Ga]Ga-Pentixafor (**g**).

[^18^F]FDG and [^68^Ga]Ga-Pentixafor revealed comparable results in 15/16 patients who underwent both [^68^Ga]Ga-Pentixafor and [^18^F]F-FDG PET/CT. The remaining patient presented with [^18^F]FDG-positive/CXCR4-negative lesions. The mean intensity of uptake in the primary lesion was higher for [^18^F]F-FDG, which is reflected by the mean SUVmax in the primary lesions of 15.7 ± 6.60 for [^18^F]F-FDG and 5.81 ± 2.74 for [^68^Ga]Ga-Pentixafor (*p*  =  0.013) as shown in Figure 2. The mean SUVmean in the tumor lesions for [^18^F]F-FDG were 9.0  ±  4.1 and 3.0  ±  1.6 for [^68^Ga]Ga-Pentixafor. When choosing the liver as a reference region, the TBR was 2.38 ± 1.47 for [^68^Ga]Ga-Pentixafor and 4.9 ± 2.2 for the [^18^F]F-FDG *p* < 0.01. There was a moderate statistically significant correlation between [^18^F]F-FDG TLU and [^68^Ga]Ga-Pentixafor TLU *r =* 0.555 *p =* 0.026.

### 3.3. Visual Analysis

**Figure 3:**
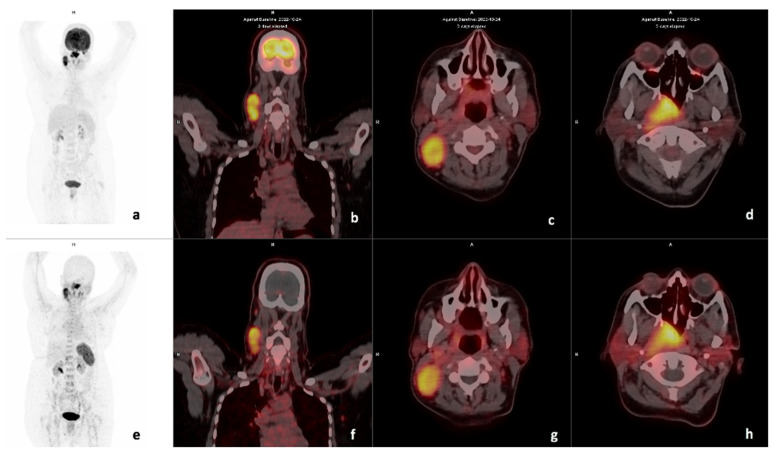
Example of a CXCR4-positive, FDG-positive patient. Display of maximum intensity projection (MIP), axial and coronal slices of both [^18^F]F-FDG PET/CT (**a**–**d**) and [^68^Ga] Ga-Pentixafor-PET CT (**e**–**h**) (interval between both scans, 3 days) in a 49-year-old female with oropharyngeal SCC (Patient #18). [^18^F]F-FDG PET/CT demonstrates intense uptake in the primary lesion (**d**) SUVmax 33.06 and right cervical lymph node (**c**) SUVmax 23.35. [^68^Ga] Ga-Pentixafor-PET also demonstrates intense tracer accumulation at the primary site (**h**) SUVmax 10.94 and cervical nodes 8.33 (**g**), respectively. CXCR4 IHC stain was strongly positive in 90% of the cells.

Lesion-based analysis showed that in 5/16 patients, the metastatic cervical lymph nodes demonstrated more avid tracer accumulation on the [^68^Ga]Ga-Pentixafor PET compared with the [^18^F]F-FDG; however, the primary lesions were more avid on the [^18^F]F-FDG than the [^68^Ga]Ga-Pentixafor PET as shown in Table 2. An example is shown in Figure 3, where the SUVmax of the left cervical lymph node is 5.29 and slightly higher than the site of primary 4.83.

**Figure 4:**
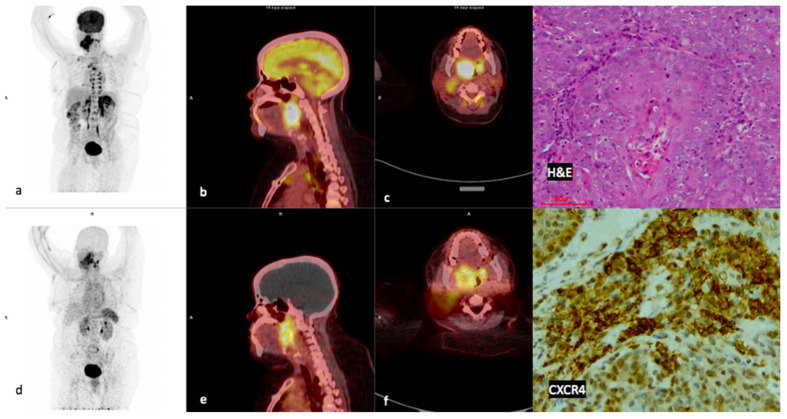
Example of a high CXCR4-positive, high FDG-positive patient. Display of maximum intensity projections of [^18^F]F-FDG (**a**) and [^68^Ga]Ga-Pentixafor (**d**) and sagittal images of both [^18^F]F-FDG-PET/CT (**b**) and [^68^Ga]Ga-Pentixafor-PET/CT (**e**) (interval between both scans, 14 days) in a 46-year-old HIV-positive female patient oropharyngeal SCC (patient #13). [^68^Ga]Ga-Pentixafor-PET (**f**) demonstrates high inhomogeneous tracer accumulation at the site of primary SUVmax 11.73 compared with 19.46 on [^18^F]F-FDG (**c**). Note the brown fat paraspinal uptake on FDG (**a**) is not present on [^68^Ga]Ga-Pentixafor (**d**). CXCR4 IHC (20× magnification) demonstrated membranous and cytoplasmic staining in 60% of the cells.

Some patients demonstrated intense uptake in brown fat on [^18^F]F-FDG; an example is shown in Figure 4, where there is intense brown fat uptake in the paraspinal region on [^18^F]F-FDG, which is not present on [^68^Ga]Ga-Pentixafor imaging.

### 3.4. Quantitative Analysis

**Figure 5:**
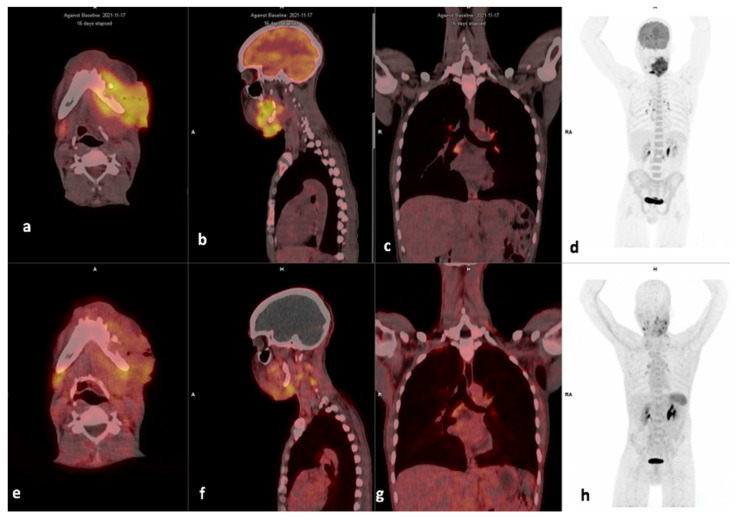
Example of a high FDG, low Pentixafor in mediastinal nodes. Display of maximum intensity projections of [^18^F]F-FDG (**d**) and [^68^Ga]Ga-Pentixafor (**h**) and axial and sagittal images of both [^18^F]F-FDG-PET/CT (**a**,**b**) and [^68^Ga]Ga-Pentixafor-PET/CT (**e**,**f**) (interval between both scans, 16 days) in a 46-year-old HIV-negative female patient oropharyngeal SCC (patient #9). [^68^Ga]Ga-Pentixafor-PET demonstrates mild-moderate inhomogenous tracer accumulation at the site of primary SUVmax 2.63 (**a**) compared with 16.93 (**e**) on [^18^F]F-FDG. Multiple foci of intense uptake are seen in inflammatory the mediastinal lymph nodes on [^18^F]F-FDG (**c**), these appear less intense on [^68^Ga]Ga-Pentixafor-PET (**g**). Notably, the spleen demonstrates higher uptake on [^68^Ga]Ga-Pentixafor (**h**) compared to [^18^F]F-FDG-PET/CT (**d**).

SUVmean in the reference regions for [^18^F]F-FDG and [^68^Ga]Ga-Pentixafor were 3.3 and 2.5 in the liver, 2.11 and 2.8 in the aorta, 3.2 and 7.2 in the spleen, reflecting the higher physiological uptake of [^18^F]F-FDG in the liver, and the higher affinity of [^68^Ga]Ga-Pentixafor to the spleen as shown in Figure 5.

### 3.5. CXCR4 Immunohistochemistry (IHC)

CXCR4 IHC staining was performed in 21/23 patients, the results are shown in Table 3. There was a moderate statistically significant correlation between CXCR4 expression and [^68^Ga]Pentixafor SUVmean (*r =* 0.5 *p =* 0.027). There was a moderate statistically significant positive correlation between CXCR4 expression and [^68^Ga]Ga-Pentixafor TLU (*r =* 0.43 *p =* 0.053). There was a strong positive correlation between CXCR4 expression and ECOG performance status (*r =* 0.83 *p =* 0.0104). A negative correlation was found between CXCR4 expression and Hb (*r =* −0.495 *p =* 0.024), as shown in Table 4. An example of a patient with high CXCR4 staining is shown in Figure 4.

### 3.6. Survival Analysis

**Figure 6:**
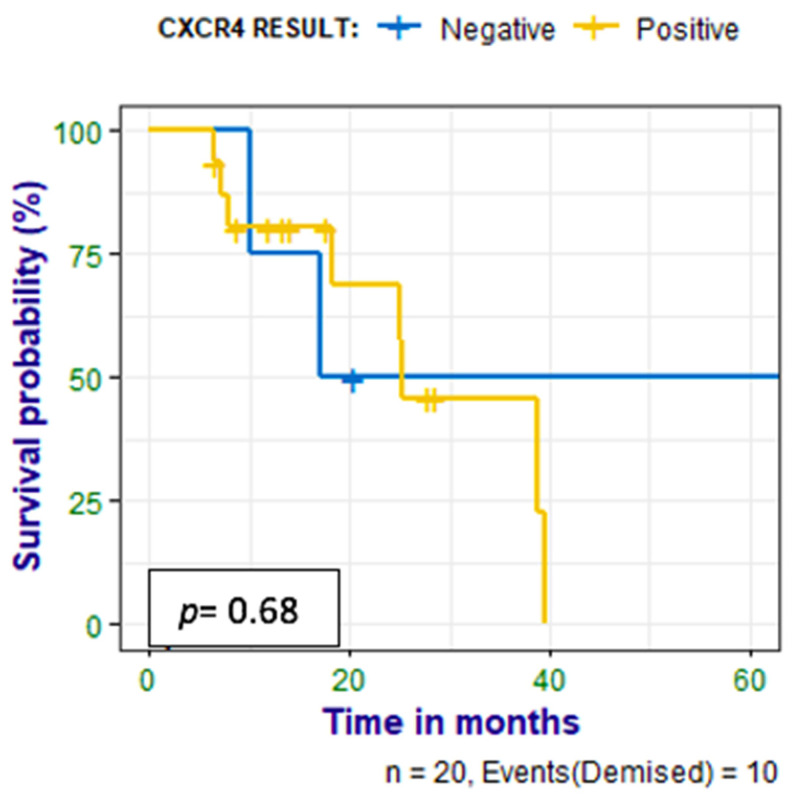
Kaplan–Meier curves show no statistically significant difference between survival in the patients who had positive IHC stains for CXCR4 and those who had negative stains.

**Figure 7:**
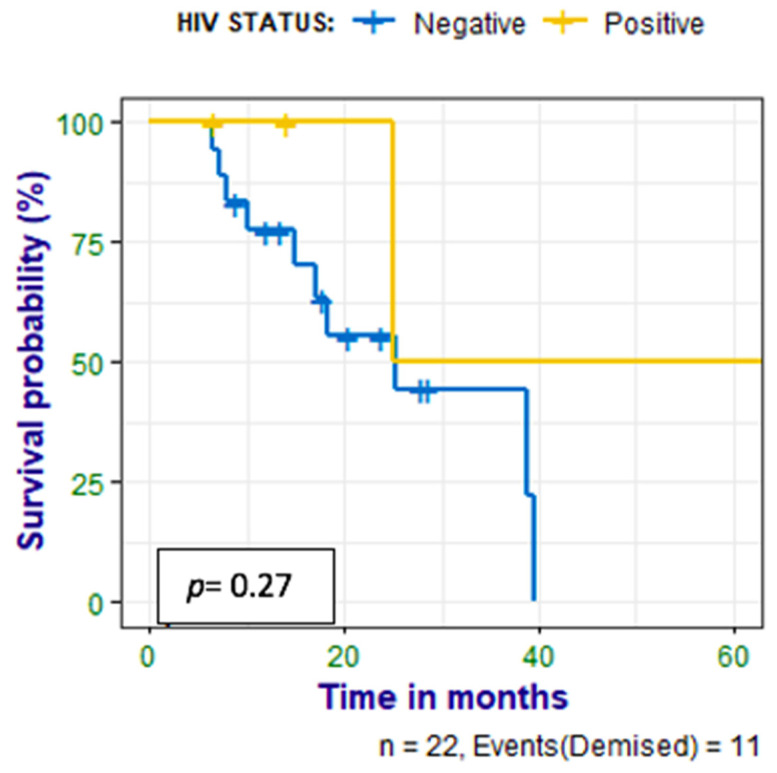
Kaplan–Meier curves show no statistically significant difference between survival in the HIV-infected patients compared with the HIV-uninfected patients with HN cancer.

Patients were followed up for 6–65 (median 17) months, and a correlation was made between survival time and CXCR4 expression, Ki-67 expression, and HIV status. Nine (41%) of the patients had died at the time of data analysis; there was no statistically significant correlation between PET metrics and survival time, as shown in Figure 6. There was a negative correlation between survival time and Ki67 (*r =* −0.560 *p =* 0.010). There was also no correlation between survival time and the HIV status of the patient as shown in Figure 7.

### 3.7. Metastasis

Distant metastases were observed on PET/CT in two patients; one patient had liver metastasis, and one had lung metastasis. The uptake was much less in the [^68^Ga]Ga-Pentixafor PET in the lung metastasis compared with [^18^F]FDG with SUVmax of 4.39 and 17.09, respectively (patient 16); however, both scans detected all the metastatic sites.

### 3.8. HPV

p16 staining was performed as a surrogate for HPV infection; six patients had positive p16 positive stains, and thirteen patients had negative stains. There was no correlation between p16 IHC staining and Pentixafor PET metrics, CXCR4 IHC staining, or survival.

### 3.9. Ki67

Ki67 staining was performed in 20/23 specimens, and one patient had negative Ki67 IHC staining. A total of 16/20 patients had high Ki67 (>50%), and only three patients had weak staining < 50%. There was a negative correlation between survival time (months) and Ki67 staining, correlation efficient *r =* −0.560 *p =* 0.010.

## 4. Discussion

To our knowledge, this is the largest cohort evaluating in vivo CXCR4 expression in HN cancer in humans. Our observation of in vivo imaging of CXCR4 expression in patients with both newly diagnosed as well as pre-treated carcinoma of the oral cavity, nasopharynx, and oropharynx confirms the presence of CXCR4 expression in HNSCC, which can be assessed non-invasively by PET/CT using the CXCR4-directed radiopharmaceutical [^68^Ga]Ga-Pentixafor. However, it should be highlighted that [^68^Ga]Ga-Pentixafor shows lower avidity when compared with [^18^F]F-FDG, similar to findings by Buck et al. [11] and Zhi et al. [12]. However, there was agreement between the [^68^Ga]Ga-Pentixafor and [^18^F]F-FDG PET metrics.

CXCR4 is upregulated in malignant diseases and has a critical role in cell survival, proliferation, angiogenesis, metastasis, migration, recurrence, and resistance to chemoradiation, which lead to tumor progression and poor clinical outcome [15,16,17]. CXCR4/CXCL12 axis is a promising therapeutic target for blocking the CXCL12/CXCR4 interaction and inhibiting downstream intracellular signaling for the treatment of various cancers [18,19,20,21]. CXCR4 inhibitors can restrict tumor cell proliferation, migration, angiogenesis, and metastasis [22]. A few classes of CXCR4 antagonists have undergone clinical trials, including peptide inhibitors, small molecule inhibitors, and monoclonal antibodies such as Plerixafor, which is approved by the Food and Drug Administration (FDA) for the treatment of non-Hodgkin’s lymphoma and multiple myeloma [23].

For all primary tumor lesions, SUVmean was lower for [^68^Ga]Ga-Pentixafor compared with [^18^F]FDG PET/CT, with an average SUVmean of 3.2 ± 1.6 for [^68^Ga]Ga-Pentixafor PET compared with 9.0 ± 4.1 for FDG. This is consistent with previous reports by Buck et al., who imaged two patients with HNSCC with [^68^Ga]Ga-Pentixafor PET [11], and Zhi et al., who compared [^68^Ga]Pentixafor and [^18^F]F-FDG in twelve patients with HNSCC [12]. When the aorta was used as the reference region, the mean ratio of the SUVmax in the primary lesion to the SUVmean within the aorta (TAR) was 10.1 for [^18^F]F-FDG and 2.16 for [^68^Ga]Ga-Pentixafor. The [^68^Ga]Ga-Pentixafor TBR is also lower than that reported by Buck et al., which was 4. The difference could be a result of a smaller number of head and neck cancers in their cohort, where only two patients had HNSCC. When the liver was used as a background, the mean TBR was 2.34 ± 1.4 compared with 4.9 ± 2.3 for [^18^F]F-FDG in the current study. Werner et al. report a higher TBR; however, they studied a heterogeneous group of patients, some of which demonstrated very high [^68^Ga]Ga-Pentixafor accumulation [24].

With regard to metastasis, the cervical lymph node metastases were more avid than the primary lesion on [^68^Ga]Ga-Pentixafor PET/CT imaging in some of the patients. This could indicate higher CXCR4 expression in the cervical node metastasis compared with the primary lesion. Muller et al. demonstrated higher CXCR4 expression in metastatic sites compared with the primary tumors in breast cancer [25]. A higher concentration of CXCL12 in lymph nodes, lung, liver, and bone/bone marrow (BM) is thought to direct the metastasis of CXCR4-expressing tumor cells [24,25]. Furthermore, [^68^Ga]Ga-Pentixafor detected more cervical nodes compared with [^18^F]F-FDG; however, histological confirmation was not performed on all of the positive nodes, and some of them may be reactive.

There was higher [^68^Ga]Ga-Pentixafor uptake in HIV-positive patients compared with HIV-negative patients. However, in the current study, only 4 of the 23 patients were HIV positive, and thus, no conclusions can be drawn on the correlation between HIV infection and CXCR4 expression in HNSCC. It has been reported that HIV infection is associated with more aggressive disease and poorer treatment outcomes in patients with HNSCC [26]. Also, there is higher CXCR4 expression in HIV as CXCR-4 also serves as a co-receptor for HIV entry into CD4+ cells [27] and is associated with more rapid immunosuppression and faster progression to AIDS [28]. Therefore, it is possible that advanced HIV infection drives CXCR-4 overexpression in cancer cells. This may be responsible for the faster tumor progression and more aggressive disease seen in HIV-infected patients with HNSCC [29]; however, this needs to be confirmed in a larger study to see if HIV-positive patients with HN cancer may benefit from therapies targeting CXCR4.

Unlike [^18^F]F-FDG, [^68^Ga]Ga-Pentixafor did not accumulate in brown fat and strained muscles, which complicates the interpretation of [^18^F]F-FDG. For example, one patient had intense physiological uptake in the salivary glands and cervical muscles on [^18^F]F-FDG, which was not present in Pentixafor (patient #4). However, similar to [^18^F]FDG, [^68^Ga]Ga-Pentixafor was not specific for malignancy and also avidly accumulated in reactive cervical lymph nodes and tonsils, which made interpretation of uptake challenging. Finally, [^68^Ga]Ga-Pentixafor accumulation was low in the majority of the patients (12/21).

Anemia has been shown to be a strong predictor of poorer survival in patients with lung carcinoma, cervix- and uterine carcinoma, head and neck carcinoma, prostate carcinoma, lymphoma, and multiple myeloma [30]. In the current study, we found a negative correlation between [^68^Ga]Ga-Pentixafor SUVmax and hemoglobin (Hb); that is, patients with anemia had a higher SUVmax on Pentixafor PET/CT. Anemia is common in cancer patients because oxygen delivery to tumors is partly regulated by the oxygen-carrying capacity of the blood and, therefore, anemia to tumor hypoxia, which is correlated with tumor aggressiveness [31].

**Figure 8:**
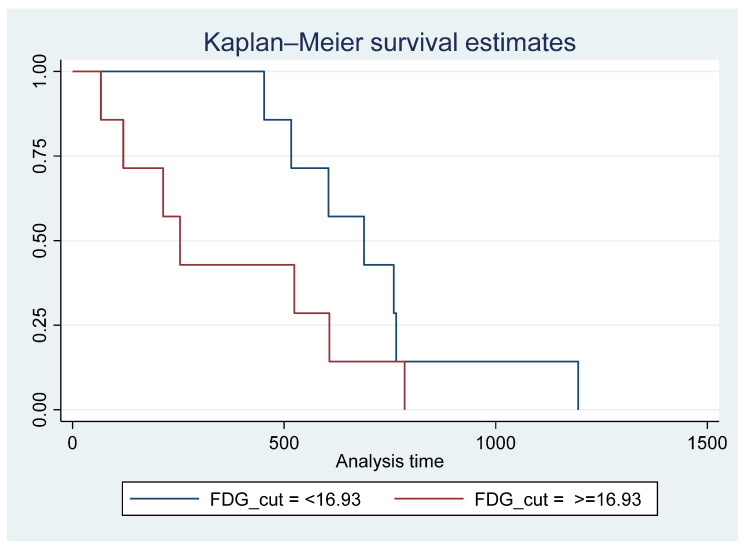
Survival time for patients with ^18^F-FDG SUVmax < 16.93 vs. >= 16.93 showing poorer survival in patients with FDG SUVmax >= 16.93; however, this did not reach statistical significance Pr > chi2 = 0.1670.

Cierpikowski et al. reported that in vitro CXCR4 was an independent prognostic factor (*p* = 0.009) [32]. In our small cohort, there was no correlation between survival and SUVmax on Pentixafor. A meta-analysis by Zhao et al. showed significantly shortened overall survival in head and neck cancer patients with CXCR4 expression (7 studies, 577 patients, HR = 2.02, 95% CI, 1.37–2.97) [15]. The discrepancy in our findings could be due to the smaller sample size. However it has been reported that [^18^F]FDG is a reliable prognostic marker in HNSCC [33,34], in the current study, patients with an FDG SUVmax of <16.93 lived longer than patients with [^18^F]F-FDG SUVmax ≥ 16.93 (Figure 8). Similarly, in a retrospective analysis of 106 previously untreated HNSCC patients, Cegla et al. showed a poorer 5-year survival in patients with FDG SUVmax of 10 compared with SUVmax of 7.7 [35].

Immunohistochemistry staining (IHC) for CXCR4 was performed in 21 of the 23 patients. There was a statistically significant moderate correlation between CXCR4 expression and [^68^Ga]Ga-Pentixafor SUVmean, TLU, as well as performance status (ECOG score). This is converse to findings by Zhi et al., where there was no correlation between [^68^Ga]Ga-Pentixafor PET metrics and in vivo CXCR4 expression. The discrepancy could be due to a smaller sample size in that study [12]. Even though we could not find any existing literature studies reporting a link between Eastern Cooperative Oncology Group performance status (ECOG-PS) scores and CXCR4 expression, it is widely known that ECOG-PS is an independent predictor of worse prognosis and indicates more aggressive disease in HNSCC patients [36].

There was a statistically significant association between CXCR4 and CXCR4 expression on IHC staining and a history of alcohol consumption. Alcohol drinkers have higher hypoxia-inducible-factor-1-alpha (HIF-1-alpha) expression in their cancer cells, which is linked with reduced survival in patients with oral cavity, pharyngeal and laryngeal cancer [37]. HIF-1-alpha is a biomarker associated with tumor invasion, metastasis, and progression, as well as angiogenesis [38]. This highlights the importance of understanding the interplay between the tumor microenvironment and environmental factors that potentiate tumor growth [39].

Despite promising results, this pilot research work has several limitations. First, the limited sample size makes it difficult to draw reasonable conclusions. Second, [^18^F]F-FDG PET/CT was not applied to all the cases due to the dismal prognosis of this condition; some of the patients died before their appointment. Third, even though there was a trend of higher Pentixafor uptake in HIV-positive HN cancer patients, only 4 of the 23 patients were HIV positive, and thus, no conclusions can be drawn on the correlation between HIV infection and CXCR4 expression in HNSCC. Lastly, this study included a heterogeneous group of head and neck malignancies, including nasopharynx, oropharynx, and oral cavity, which are known to behave differently.

## 5. Conclusions

In conclusion, although [^68^Ga]Ga-Pentixafor cannot replace [^18^F]F-FDG as a diagnostic tool because of its lower avidity, the correlation between CXCR4 targeted ^68^Ga-Pentixafor PET imaging and CXCR4 IHC staining indicates the potential of ^68^Ga-Pentixafor as an effective tool for selecting patients who may benefit from therapies targeting CXCR4. In addition, [^68^Ga]Ga-Pentixafor has no physiological brown fat uptake, which often obscures cervical lesions on [^18^F]F-FDG PET/CT imaging.

## Figures and Tables

**Table 1:** Demographic and clinical characteristics of participants.

Patient No.	Age	Gender	Site	HIV Status	TNM	Differentiation	Histology
1	61	Male	oropharynx	Negative	T4aN2cM0	moderate	SCC
2	51	Female	Oral cavity	Negative	T4aN3bM0	poor	SCC
3	72	Male	oropharynx	Negative	T4N1M0	moderate	SCC
4	47	Female	Oral cavity	Negative	T4aN2cM0	moderate	SCC
5	49	Male	Unknown primary	Positive	TxN3bM0	moderate	SCC
6	60	Male	oral cavity	Negative	T4acN3bM0	moderate	SCC
7	50	Male	oral cavity	Negative	T4aN2cM0	moderate	SCC
8	51	Female	oral cavity	Negative	T3N1M0	moderate	SCC
9	59	Male	oral cavity	Negative	T4N3bM0	moderate	SCC
10	68	Male	oropharynx	Negative	T4bN1M0	myoepithelial ca	myoepithelial
11	53	Male	oropharynx	Negative	T4aN1M0	moderate	oropharynx
12	73	Male	oral cavity	Negative	T3N0M0	moderate	SCC
13	46	Female	oropharynx	Positive	T3N3bM0	moderate	SCC
14	48	Male	oral cavity	Negative	T4bN2cM0	moderate	SCC
15	39	Male	oropharynx	Positive	T4bN0M1	mucoepidermoid	mucoepidermoid
16	52	Male	nasopharynx	Negative	T4N2M1	poor	SCC
17	42	Male	oropharynx	Negative	T1N3bM0	moderate	SCC
18	49	Female	nasopharynx	Negative	T1N1M0	undifferentiated	SCC
19	35	Male	oropharynx	Negative	T3N0M0	moderate	SCC
20	57	Male	oral cavity	Negative	T3N0M0	moderate	SCC
21	61	Male	oral cavity	Negative	T4aN0M0	moderate	SCC
22	68	Female	oral cavity	Negative	T4aN2cM0	moderate	SCC
23	46	Male	oropharynx	Positive	T4N3M1	moderate	SCC

**Table 2:** PET metrics of primary tumors.

Patient No	SUVmax [g/mL]		SUVmean [g/mL]		Tumour:Aorta Ratio		Total Lesion Uptake [cm^3^]		Likert Scale		Visual *	
	FDG	Pentixafor	FDG	Pentixafor	FDG	Pentixafor	FDG	Pentixafor	FDG	Pentixafor	FDG	Pentixafor
1	11.9	3.1	6.9	2.0	4.6	1.1	98.2	46	5	4	5	1
2	8.9	5.0	4.9	2.3	6.3	2.3	129	66	5	5	5	0
3	8.2	3.5	5.1	2.3	9.3	1.4	34.1	60	5	5	5	1
4	18.9	3.7	11.6	2.4	7.0	1.6	124	82	5	4	2	2
5	15.3	5.6	8.0	3.4	5.7	1.4	477	477	5	4	5	4
6	18.4	4.8	10.3	3.0	10.7	2.2	277	136	5	4	5	3
7	13.5	3.4	7.5	1.8	9.7	1.6	49	98	5	3	5	2
8	-	2.8	-	1.7	-	1.2	-	30	5	4	-	0
9	16.9	5.6	8.8	2.8	10.8	2.4	906	288	5	5	5	3
10	13.0	8.4	7.6	2.9	4.6	2.1	367	170	5	5	5	3
11	-	8.6	-	4.5	-	2.7	-	312	-	5	-	2
12	-	4.9	-	1.2	-	1.6	-	76	-	4	-	4
13	19.5	11.7	11.9	6.7	8.0	2.8	402	470	5	5	5	4
14	9.8	3.2	6.4	2.0	5.7	1.2	13.3	16	5	4	5	1
15	21.7	4.9	11.7	3.0	9.4	1.6	204	137	5	4	5	2
16	-	8.9	-	4.9	-	2.8	-	34	-	4	-	4
17	-	5.3	-	0.8	-	1.6	-	10	-	3	-	3
18	33.1	10.9	20.7	6.5	10.1	6.4	236	66	5	5	5	5
19	-	8.2	-	4.0	-	2.4	-	49	-	5	-	5
20	-	4.6	-	2.5	-	2.2	-	301	-	0	-	4
21	50.5	5.1	29.7	2.1	16.0	1.8	393	27	5	0	5	1
22	60.0	7.1	36.3	3.1	19.2	1.6	1240	269	-	4	5	4
23	20.2	6.0	12.2	6.0	9.0	2.1	531	162	5	-	5	2

* 5 point Likert scale: 0 = no uptake, 1 = very mild, 2 = mild, 3 = mild-moderate, 4 = moderate, 5 = intense.

**Table 3:** Immunohistochemistry results.

Patient No.	Ki67	p16	CXCR4			^68^Ga-Pentixafor		
			Intensity	% Stained Cells	IRS Scoring	TMR	TSR	TLR
1	70%		3	50%	10	1.83	0.5	1.29
2						4.96	0.7	2.23
3	70%	40%	++	40%	4	3.76	0.4	1.31
4	50%		+++	50%	6	3.04	0.5	1.95
5	90%		+++	90%	12	2.34	0.6	1.55
6						3.64	0.9	2.81
7	60%	Negative	+	20%	2	2.41	-	1.99
8	30%	70%	+++	40%		3.47	0.5	1.34
9	10%	negative	++	30%	4	3.53	1.4	2.63
10	40%	negative	++	40%	4	4.08	0.8	2.78
11	70%	50%	++	70%	6	3.73	1.5	3.03
12	80%	60%		0	0	4.22	0.6	1.79
13	90%	95%	++	60%	6	6.90	1.4	3.48
14	95%	negative	negative	0	0	2.32	0.3	1.03
15	90%	negative		0	0	4.81	0.7	1.85
16	90%	negative	+++	60%	9	6.30	1.0	-
17	60%	10%	0	0	0	4.19	0.6	1.68
18	70%	negative	2	10%	4	17.37	1.9	7.60
19	8%	20%	+++	80%	9	5.91	1.0	2.51
20	40%	3%	+++	70%	9	3.70	0.9	2.35
21	60%	Negative	Negative	0	0	4.25	0.8	2.07
22	60%	Negative	++	5%	2	5.51	0.6	1.35
23	95%		++	30%	2	5.56	1.1	3.01

IRS: Immunoreactive score; + mild, ++ moderate, +++ intense; 0–1 negative, 2–3 mild, 4–8 moderate, 9–12 strongly positive. CXCR4 Immunohistochemistry.

**Table 4:** Correlation between CXCR4 IHC staining with Age, N stage, PET/C parameters, Ki67, and Hb.

Variable	Correlation Co-Efficient	*p* Value
Age	−0.057	0.812
N stage	0.1	0.657
M stage	−0.048	0.841
[^68^Ga]Ga-Pentixafor SUVmax	0.351	0.129
[^68^Ga]Ga-Pentixafor TMR	0.096	0.686
[^68^Ga]Ga-Pentixafor TSR	0.22	0.357
[^68^Ga]Ga-Pentixafor SUVmean	0.5	0.027 *
[^68^Ga]Ga-Pentixafor TLU	0.43	0.053 *
[^18^F]FDG SUVmax	−0.202	0.490
[^18^F]FDG TLU	0.180	0.539
[^18^F]FDG SUVmean	−0.185	0.526
Ki67	0.317	0.174
Hb	−0.495	0.024 *
ECOG score	0.83	0.0104 *

TMR: Tumor-to-muscle ratio, TSR: Tumor-to-spleen ratio, TLU: Total lesion uptake, ECOG: Eastern Cooperative Oncology Group. * statistically significant.

## Data Availability

The raw data supporting the conclusions of this article will be made available by the authors on request.

## Abbreviations

CXCR4	Chemokine receptor 4
FDG	Fluorodeoxyglucose
HNSCC	Head and neck squamous cell carcinoma
HPV	Human Papilloma virus
IHC	Immunohistochemistry
RT	Radiation therapy
TBR	Tumor-to-background ratio
TLU	Total Lesion Uptake
